# Combined Neonatal Alloimmune Neutropenia and Thrombocytopenia in Dizygotic Twins Conceived After Oocyte Donation

**DOI:** 10.7759/cureus.35950

**Published:** 2023-03-09

**Authors:** Carolina Folques, Beatriz de Sá, Margarida Agostinho, João do Agro, António Martinho, Gabriela Rangel, Joana Marques, Joana Azevedo

**Affiliations:** 1 Pediatric Hematology Unit, Department of Clinical Hematology, Hospital and University Centre of Coimbra, Children's Hospital of Coimbra, Coimbra, PRT; 2 Department of Pediatrics, Leiria Hospital Centre, Leiria, PRT; 3 Blood and Transplant Center of Coimbra, Instituto Português do Sangue e Transplantação, Coimbra, PRT; 4 Blood and Transplant Center of Porto, Instituto Português do Sangue e Transplantação, Porto, PRT

**Keywords:** hla class i, hpa-5b, neonatal alloimmune thrombocytopenia, alloimmune neonatal neutropenia, thrombocytopenia, neutropenia

## Abstract

Neonatal alloimmune thrombocytopenia (NAIT) and neonatal alloimmune neutropenia (NAIN) may have severe consequences in the neonatal period. We report two dizygotic twins conceived after donated oocytes, suffering NAIT and NAIN in the context of alloantibodies to human platelet antigens (anti-HPA-5b) and human leukocyte antigens (anti-HLA class I). Genotyping demonstrated paternal homozygosity for HPA-5a, while the neonates were heterozygous for HPA-5b.

## Introduction

Neonatal alloimmune thrombocytopenia (NAIT) and neonatal alloimmune neutropenia (NAIN) are caused by maternal exposure to paternally inherited human platelet antigens (HPA) and human neutrophil antigens (HNA), respectively, expressed on the surface of fetal platelets and neutrophils that cross the placenta [1-3].

Alloantibodies formation (IgG) is a common and usually innocuous event during pregnancy, but when transported across the placenta to fetal circulation these alloantibodies may cause destruction of platelets and neutrophils, placing the neonate at risk of developing severe cytopenias [3-4].

Incidence of NAIT has been estimated at 1 in 1000 to 10000 live births [5-7], while the incidence of NAIN has been reported to be between 2/1000 to 8/10000 live births (some studies have estimated lower rates up to 1/120000) [2,8-11].

The genetic systems of antigens found at platelets and neutrophils surface are known as HPA and HNA, respectively. Platelet antigens, expressed on different platelet glycoproteins, are currently grouped in 35 HPA systems, labelled from HPA-1 to HPA-35. In European populations, alloantibodies are more commonly developed against antigens within the HPA-1, HPA-2, and HPA-5 systems [12]. Similarly, the neutrophil antigens are classified in five distinct HNA systems (HNA-1, 2, 3, 4 and 5), known to encode glycoproteins responsible for neutrophil adhesion and function. HNA-1 is apparently more immunogenic, causing most of NAIN cases [2].

The frequency of human antigens systems varies according to ethnic distribution and among different geographical areas, influencing the formation of specific antibodies and incidence of alloimmune disorders [12].

Among alloantigens of platelet and neutrophil membrane shared with other cells are the glycoconjugates of the ABO system and human leukocyte antigens (HLA) [13]. The latter are divided into class I genes (loci A, B and C) and class II genes (loci DR, DQ and DP).

HLA antibodies have been implicated as an etiology for NAIT and NAIN [14]. Nonetheless, the correlation between anti-HLA alloantibodies and neonatal thrombocytopenia and neutropenia is extremely rare, in part because of reduced expression of HLAs in neonatal cells. A limited number of reports have implicated the transplacental passage of maternal HLA antibodies as a cause of NAIT and NAIN [14].

We report a case of two dizygotic twins conceived after donated oocytes, who suffered neonatal thrombocytopenia and neutropenia, in the context of alloantibodies anti-HPA-5b and anti-HLA class I detected on maternal serum. Platelet antigen genotyping showed homozygosity for both parents (HPA-5a), while the two neonates revealed to be heterozygous, carrying both HPA-5a/b.

## Case presentation

Two dizygotic twins (female and male) were late preterm, born at 36 weeks by elective cesarean delivery (due to previous cesarean), with a birthweight of 2400g and 2600g (appropriate for gestational age). Apgar score was 9 and 10 at 1 and 5min respectively, for both newborns. The pregnant surrogate mother was a Caucasian 45-year-old woman, gravida 2, para 1, who underwent a first in vitro fertilization using donated oocytes and father sperm. Father was a Caucasian man, 44 years old, with no relevant medical history. There was no maternal use of prescription medications before or during pregnancy and no history of blood transfusions, surgeries, or severe trauma. She had a 15-year-old daughter, from a different father, with Congenital Muscular Dystrophy with merosin deficiency. The remaining family history was noncontributory. The current dichorionic-diamniotic twin pregnancy was complicated by gestational diabetes controlled by diet. Maternal serological screening for hepatitis B, HIV and syphilis was negative, and she demonstrated immunity to Toxoplasma gondii and Rubella virus. Cytomegalovirus serologic screening was not performed. Prenatal ultrasound showed no anomalies. Routine screening for Group B Streptococcus was negative. Rupture of the membranes occurred 7 hours before delivery.

The first twin (female) displayed feeding difficulties shortly after delivery. Hypoglycemia was detected 2 hours after birth, sustained at 4 and 6 hour (minimum 22 mg/mL), refractory to enteral feeding and oral 10% glucose solution (2 ml/kg). This newborn developed scattered petechiae and one suffusion along the sternal region, 24 hours after birth. A complete blood count performed at 36 hour of life revealed a white blood cell count of 5100/μL, absolute neutrophil count (ANC) of 1600/μL, hemoglobin of 9.4 g/dL, and platelet count of 13,000/μL. The second twin (male), though clinically well, was also investigated, revealing a normal white blood cell count, but hemoglobin of 12.8 g/dL and platelet count of 12,000/μL. C-reactive protein (CRP) was normal for both newborns. Lactate dehydrogenase and reticulocyte indexes were elevated (702 and 744 U/L, 3.2 and 3.0%, respectively), and haptoglobin and bilirubin were within the reference range. Direct Coombs testing was negative. Examination of the peripheral smears revealed anisopoikilocytosis in both. The surrogate mother’s blood group was O Rh(D) positive, with a negative direct antiglobulin test and irregular antibody screenings. Her cell blood count (CBC) was normal throughout pregnancy with Hb 10.8 g/dL, ANC 11600/μL, and a platelet count of 145,000/μL 24 hours after delivery.

The first infant was presumed septic and placed on empiric antibiotics (ampicillin and gentamicin). Viral etiology workup (including Cytomegalovirus, Parvovirus B19 and HHV-6 Real-Time quantitative Polymerase Chain Reaction) and peripheral blood cultures were negative. Transfontanellar ultrasound showed no intracranial hemorrhage, and no other relevant findings.

**Figure 1:**
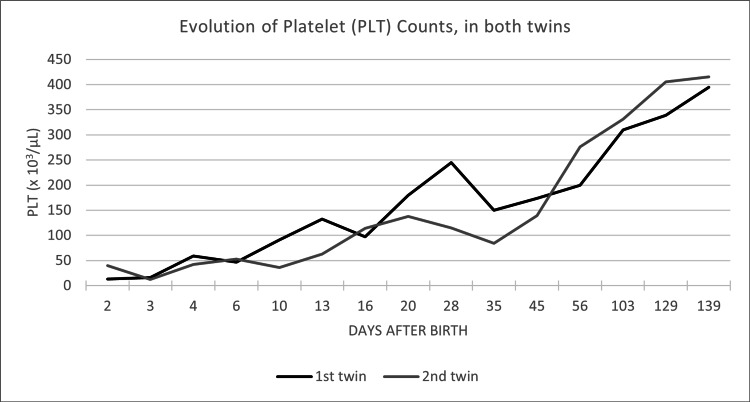
Evolution of Platelet (PLT) Counts, in both twins Platelet count evolution, in the 1st and 2nd twins.

The newborns received two platelet transfusions from random donors (15 ml/kg) (Figure 1), as HPA-matched platelets were not available at that time, with no improvement in the platelet count within hours after transfusion, suggesting immune-mediated platelet destruction. They were given two doses of intravenous immunoglobulin (IVIg) (1 g/kg) over two consecutive days (total dose of 2 g/kg).

**Figure 2:**
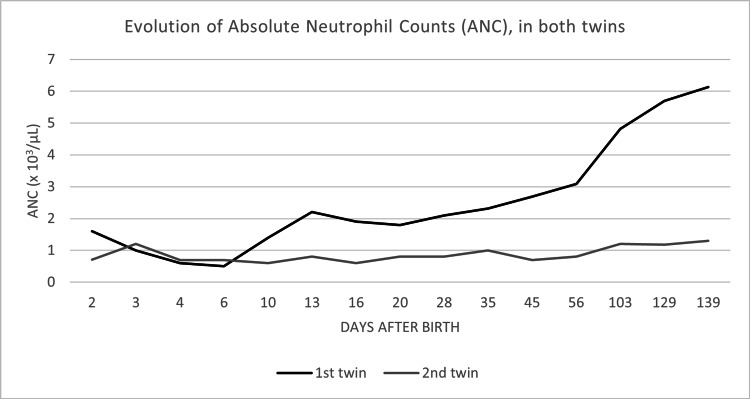
Evolution of Absolute Neutrophil Counts (ANC), in both twins Absolute neutrophil count evolution, in the 1st and 2nd twins

On the fourth day of life, the ANC of both twins was found to be low, with 600/μL and 700/μL in the first and second twins, respectively; nadirs of 500/μL and 600/μL in the serial controls (Figure 2). Absolute counts of T, B and NK cells were normal, analyzed by flow cytometry.

**Figure 3:**
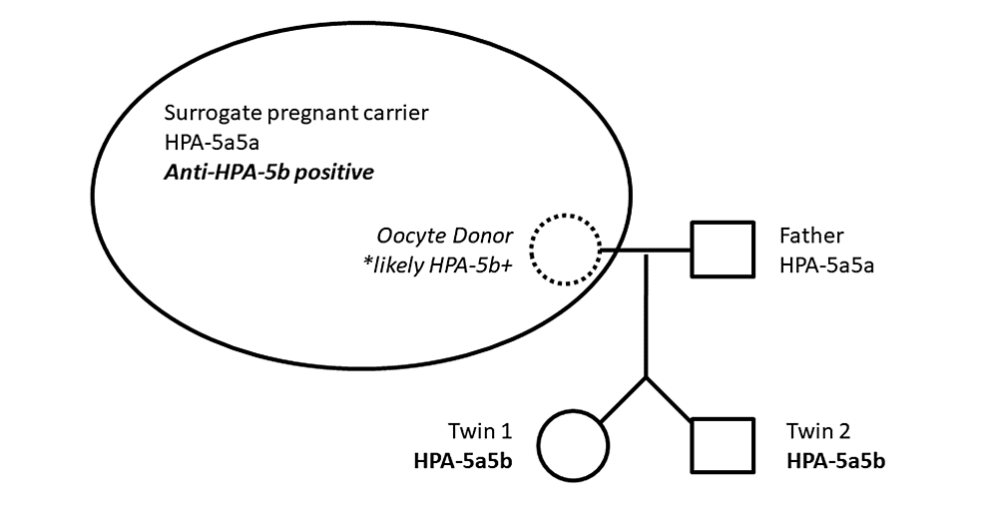
HPA antigen genotyping in the family Graphic representation of the HPA-5 system genotype in the family. * Oocyte donor genotype was not performed, but she was presumed to be a carrier of the HPA-5b antigen due to its presence in both twins, and absence in the father. HPA: Human platelet antigens

To further investigate neutropenia and thrombocytopenia, screening for anti-HNA, anti-HPA and anti-HLA antibodies was performed on maternal and neonates’ sera, by enzyme-linked immunosorbent assay (ELISA). The screening detected maternal antibodies directed against HPA-5b and HLA class I. Antibodies to HNA (HNA-1A, 1B, 1C, 2, 3A, 3B, 4A, 5A and 5B) were not detected. Cross-matching between the surrogate mother’s serum and father’s platelets was strongly positive. A complementary platelet antigen genotyping study showed both surrogate mother and father were homozygous for HPA-5a, while the two neonates were heterozygous (HPA-5a/b) (Figure 3).

The first twin received a red blood cell transfusion for acute anemia with hemoglobin of 6.8 g/dL, at two weeks old, with rapid correction of hemoglobin concentration. Both received iron and folic acid supplementation. Platelet count was normalized at 20 and 45 days old (Figure 1), while the resolution of neutropenia occurred at 13 days and 3 months old, in the first and second twins respectively (Figure 2). None of the newborns received granulocyte colony-stimulating factor (G-CSF). Their CBC remained normal at revaluation at 4 and 8 months old.

## Discussion

These dizygotic twins’ thrombocytopenia and neutropenia presentation and evolution strongly suggested a shared alloimmune process (age of presentation, mild symptoms, evolution of cell counts, platelet transfusion refractoriness, spontaneous recovery within a few months).

The presence of alloantibodies against HPA, HLA and later HNA was thus investigated, detecting maternal antibodies against HPA-5b and HLA class I. Neonates and parental platelet antigen genotyping surprisingly disclosed homozygosity in both the surrogate mother and father for HPA-5a, while the neonates were heterozygous for HPA-5a/b. Based on these results, we consider that the oocyte donor could likely carry the HPA-5b allele, inherited by both fetuses, inducing surrogate pregnancy carrier sensitization following transplacental passage of platelets. The identification of circulating alloantibodies developed by the pregnancy carrier mother (“surrogate”, genotyped as HPA-5a5a) directed against the HPA-5b antigen, present in both newborns’ platelets, could thus account for their early onset neonatal thrombocytopenia.

Regarding the involvement of HPA antibodies, anti-HPA-1a has been described as the most prevalent in pregnant women, followed by anti-HPA-5b. However, given their high prevalence in women delivering healthy newborns, the association with NAIT has been hypothesized to be coincidental. Alm et al. [15] showed evidence that a proportion of pregnant women presenting with anti-HPA-5b antibodies will give birth to a newborn with mild thrombocytopenia. In another study, Ohto et al. [16] demonstrated an association between high titer of anti-HPA-5b and a higher risk of neonatal thrombocytopenia, even if severe thrombocytopenia rarely develops.

Although improbable, the presence of antibodies against other less frequent or novel HPA, HLA or HNA antigens could not be completely ruled out. Knowing that HLA-I proteins are expressed on neutrophils and platelets, HLA-I antibodies could be the immunological cause for the neutropenia in both twins, or even contribute to both cytopenias. The presence of HLA antibodies is relatively common in many healthy pregnancies, with a prevalence of 10-30% if nulliparity [17] increasing with parity up to 74%, in women who have had more than two deliveries [18]. However, reports of HLA antibody-induced NAIN and/or NAIT are rare. This inconsistency could be explained by selectivity in transplacental crossing of HLA antibodies, reduced HLA expression on neonatal cells, or binding of alloantibodies to neonatal macrophages resulting in inhibition of cell destruction [14,18].

Oocyte donation (OD) pregnancies represent a unique situation with a higher level of antigenic dissimilarity between mother and fetus compared to spontaneously conceived pregnancies. Therefore, it has been demonstrated a higher number of HLA mismatches and a greater extent of maternal humoral immune response in OD pregnancies, with increased incidence of child-specific HLA antibody formation [17]. Additionally, the enhanced maternal age (45 years, in this case report) could possibly enable a longer period for immunizing events such as unrecognized pregnancies to occur. On the other hand, twin pregnancies are associated with higher volumes of feto-maternal hemorrhage and enlarged exposure to fetal antigens, positively correlated with the presence of HLA antibodies [17].

Reports of HLA antibody-induced NAIN and/or NAIT are characterized by the absence of anemia, as mature erythrocytes do not present HLAs. In this report, both neonates developed anemia, the first presenting with hemoglobin 9.4 g/dL (36 hours after birth) and requiring transfusion at 2 weeks old. We hypothesize this could be a multifactorial anemia due to an element of undetected feto-placental/maternal transfusion, early cord clamping, umbilical blood sampling and nutritional deficiencies (iron and folate) at birth, probably exacerbating their already higher vulnerability as preterm and low-birth-weight infants, aggravated by the inflammatory disease component of her presumed early septic complication. The heavy laboratory phlebotomy loss, sustained shortly and repeatedly after birth, probably worsened these pre-existing conditions [13-15]. Other non-physiological causes of anemia (such as active or internal hemorrhage, or immune-mediated hemolysis) were ruled out during the investigation, and her hemoglobin levels remained stable afterwards.

As with other neonatal alloimmunologic processes, passive antibody clearance resulted in complete recovery of platelet counts to normal within two months of life and resolution of neutropenia within three months. HLA antibody testing was not repeated, since there was no recurrence of cytopenias, supporting the expected decline of maternal antibodies in the infant’s serum.

## Conclusions

Different screening techniques and variability in screening time points have resulted in discrepancies regarding the reported investigation of alloimmune cytopenias, particularly in the neonatal period. More detailed studies on the incidence, characteristics and specificity of the antibodies involved are essential. Considering this is a potentially preventable pathology, associated with increased neonatal and even fetal morbimortality, we point up the need for further research, particularly on the possible epidemiologic impact of including HPA (or even HLA) typing in screening programs for gamete and embryo donation, matching donors and recipients, particularly in multiparous mothers/surrogates.

Considering maternal transfer of alloantibodies as the cause of neonatal cytopenias requires the exclusion of other etiologies, along with a high suspicion index. NAIN and NAIT pose a risk to newborns’ life, therefore prompt and specific approaches to diagnosis and clinical management are required to ensure appropriate support (such as antigen-negative donor platelets, in NAIT) as soon as possible.
